# Analysis of factors influencing clinical pregnancy rates in frozen-thawed embryo transfer cycles

**DOI:** 10.3389/fendo.2025.1551530

**Published:** 2025-06-25

**Authors:** Junqiang Wang, Zexing Yang, Ying Chen, Fengchen Gao, Wenxiu Zhao, Shuxuan Cao, Yixi Li, Limei He

**Affiliations:** ^1^ School of Public Health, Kunming Medical University, Kunming, China; ^2^ Department of Reproductive Genetics, The First Affiliated Hospital of Kunming Medical University, Kunming, China; ^3^ Department of Epidemiology and Health Statistics, School of Public Health, Kunming Medical University, Kunming, China

**Keywords:** assisted reproductive technology, frozen-thawed embryo transfer, clinical pregnancy, female age, influencing factors

## Abstract

**Objective:**

To identify the determinants influencing clinical pregnancy outcomes in frozen-thawed embryo transfer (FET) cycles.

**Methods:**

A retrospective analysis was conducted on patients who underwent FET at the Department of Reproductive Genetics, The First Affiliated Hospital of Kunming Medical University, between January 2018 and December 2023. A total of 7,302 FET cycles were included and categorized into two groups based on clinical pregnancy outcomes: the clinical pregnancy group (n = 3,365) and the non-clinical pregnancy group (n = 3,937). Baseline characteristics were compared between groups. A random forest algorithm was applied to rank the importance of variables, followed by dimensionality reduction using a sliding window sequential forward selection (SWSFS) method. The top-ranked predictors with the lowest average out-of-bag (OOB) error rates were incorporated into a multivariate logistic regression model to determine independent predictors of clinical pregnancy in FET cycles.

**Results:**

The overall clinical pregnancy rate (CPR) was 46.08%. The CPR was significantly higher in blastocyst transfers (61.14%) compared to cleavage-stage embryo transfers (34.13%) (χ² = 528.973, *P* < 0.01). The random forest model identified seven variables with the highest predictive value: female age, number of high-quality blastocysts, anti-Müllerian hormone (AMH) level, embryo stage at transfer, endometrial thickness on the day of transfer, number of high-quality cleavage-stage embryos, and pre-transfer endometrial thickness. Multivariate logistic regression analysis demonstrated that younger female age (OR: 0.93; 95% CI: 0.92–0.94), greater number of high-quality blastocysts (OR: 1.67; 95% CI: 1.49–1.88), higher AMH levels (OR: 1.03; 95% CI: 1.01–1.05), blastocyst transfer (OR: 2.31; 95% CI: 1.85–2.88), increased endometrial thickness on transfer day (OR: 1.10; 95% CI: 1.05–1.15), more high-quality cleavage-stage embryos (OR: 1.74; 95% CI: 1.59–1.92), and greater pre-transfer endometrial thickness (OR: 1.04; 95% CI: 1.00–1.09) were all independently associated with higher clinical pregnancy rates.

**Conclusion:**

Female age, number of high-quality blastocysts, AMH levels, embryo stage at transfer, endometrial thickness on the day of transfer, number of high-quality cleavage-stage embryos, and pre-transfer endometrial thickness are significant predictors of clinical pregnancy outcomes in FET cycles. These findings may guide individualized embryo transfer strategies to optimize reproductive success.

## 1 Introduction

With the ongoing development of socioeconomic conditions and the evolving landscape of women’s career planning, delayed marriage and childbearing have become increasingly common. However, as female age advances, female fecundity tends to decline, leading to a higher prevalence of infertility. The World Health Organization (WHO) defines infertility as a disease of the reproductive system, characterized by the failure to achieve a clinical pregnancy after 12 months or more of regular unprotected sexual intercourse. Epidemiological data indicate a significant global increase in infertility prevalence between 1990 and 2021, with East Asia exhibiting the highest rates (1). In some low- and middle-income countries, particularly in sub-Saharan Africa, infertility prevalence has reached alarming levels of 30%–40% (2, 3).

In China, findings from four successive national reproductive health surveys reveal a rising trend in infertility prevalence, increasing from 11.9% in 2007 to 17.6% in 2020 (4). In response to this growing public health concern, assisted reproductive technology (ART) has emerged as a critical intervention to support individuals and couples in achieving their reproductive goals. Among various ART procedures, frozen-thawed embryo transfer (FET) has become an essential approach, particularly beneficial for patients who are unsuitable for fresh embryo transfer, such as those of advanced maternal age, elevated serum progesterone levels, intrauterine fluid accumulation, or at risk for ovarian hyperstimulation syndrome (OHSS). FET is widely applied in clinical practice due to its capacity to reduce complications and improve cumulative pregnancy outcomes (5–7).

Clinical pregnancy represents the initial and fundamental indicator of ART success. However, outcomes following FET are influenced by a multitude of factors, including maternal age, endometrial preparation protocols, endometrial thickness on the day of embryo transfer, and the type of embryo transferred (8, 9). Identifying these determinants and implementing precise, individualized interventions to optimize clinical pregnancy rates (CPRs) has become a focal point in reproductive medicine research.

Random forest (RF), a widely used machine learning algorithm, offers advantages such as resistance to overfitting, robustness against multicollinearity, and the ability to rank the relative importance of a large number of predictors. Meanwhile, binary logistic regression models provide intuitive interpretations of associations through odds ratios (ORs), complementing RF’s limitations in this regard.

In this study, we conducted a retrospective analysis of 7,302 FET cycles, integrating RF and binary logistic regression models to comprehensively explore the factors influencing clinical pregnancy outcomes in FET cycles. Our findings aim to contribute valuable evidence toward improving CPRs, thereby reducing the psychological and financial burdens on infertile couples.

## 2 Materials and methods

### 2.1 Clinical data collection

This retrospective study included clinical data from patients who underwent frozen-thawed embryo transfer (FET) cycles at the Department of Reproductive Genetics, The First Affiliated Hospital of Kunming Medical University, between January 2018 and December 2023. The collected variables encompassed female age, female body mass index (BMI), male BMI, type of infertility (primary or secondary), duration of infertility, endometrial preparation protocol, serum anti-Müllerian hormone (AMH) levels, fertilization method in the fresh cycle, endometrial thickness prior to transfer, embryo type transferred (cleavage-stage or blastocyst), number of high-quality blastocysts, number of non-high-quality blastocysts, number of high-quality cleavage-stage embryos, number of non-high-quality cleavage-stage embryos, endometrial thickness on the day of transfer, and clinical pregnancy outcome.

A total of 7,302 FET cycles were included in the analysis. Based on the clinical pregnancy outcome, cycles were categorized into two groups: the clinical pregnancy group (n = 3,365; 46.08%) and the non-clinical pregnancy group (n = 3,937; 53.92%).

This study was approved by the Ethics Committee of Kunming Medical University (Approval No. KMMU2023MEC020). All participants provided written informed consent after being fully informed about the study procedures and objectives.

### 2.2 Inclusion and exclusion criteria

Inclusion criteria were as follows: (1) Patients who met the clinical indications for frozen-thawed embryo transfer (FET) and had no severe organic or immunological diseases; (2) Couples whose infertility was attributed to female tubal or pelvic factors, ovulatory dysfunction, or male factor infertility.

Exclusion criteria included: (1) Patients diagnosed via B-mode ultrasound with endometriosis, endometrial polyps, or submucosal fibroids; those with congenital reproductive tract anomalies; and cases in which either partner had known chromosomal abnormalities; (2) Patients whose FET cycles were canceled due to ovarian hyperstimulation, inadequate endometrial development, or other medical reasons; (3) Individuals with incomplete essential clinical data or those lost to follow-up.

### 2.3 Embryo grading criteria

Embryos were graded based on the consensus guidelines established by Chinese reproductive medicine experts (10). For analytical purposes, embryos were categorized into two groups: high-quality embryos—including high-quality cleavage-stage embryos and high-quality blastocysts—and non-high-quality embryos, which comprised all remaining cleavage-stage embryos and blastocysts not meeting the high-quality criteria.

Cleavage-stage embryos were evaluated according to the number and uniformity of blastomeres, the presence of multinucleation, and the proportion of cytoplasmic fragments. Based on these morphological criteria, embryos were classified into four grades: Grade I, II, III, and IV. Grade I and II embryos, which demonstrate optimal developmental potential and higher implantation rates, were designated as high-quality cleavage-stage embryos. Grade III embryos were considered usable but suboptimal, while Grade IV embryos were deemed non-viable for transfer.

Blastocyst grading was performed using a comprehensive evaluation of three parameters: the degree of blastocoel expansion, the quality of the inner cell mass (ICM), and the quality of the trophectoderm (TE). The expansion stage was graded on a 1–6 scale, where stages ≥3 were considered sufficiently developed for further assessment. The ICM was graded as A, B, C, or D, and the TE as A, B, or C. Blastocysts with an expansion stage of ≥3 and both ICM and TE grades of B or higher were classified as high-quality blastocysts.

### 2.4 Outcome assessment

Serum β-human chorionic gonadotropin (β-hCG) levels were measured 12–14 days after embryo transfer. A serum β-hCG concentration ≥5 IU/L was defined as a positive biochemical pregnancy. Transvaginal ultrasound was subsequently performed 28–35 days post-transfer to assess for clinical pregnancy. Clinical pregnancy was diagnosed by the presence of an intrauterine gestational sac on ultrasound, or the identification of chorionic villi in extrauterine tissue in cases suggestive of ectopic implantation.

The primary outcome measure was the clinical pregnancy rate (CPR).

### 2.5 Quality control

Data entry was performed using a double-entry system. After every 150 records were entered, data verification and logical consistency checks were conducted to promptly identify and rectify errors. Cases with incomplete data were excluded in a timely manner to ensure data integrity.

### 2.6 Statistical analysis

Data preprocessing and statistical analyses were conducted using R software version 4.4.1. Continuous variables with a normal distribution were expressed as mean ± standard deviation (SD), whereas non-normally distributed continuous variables were presented as median (interquartile range, *P*_25_–*P*_75_). Categorical variables were summarized as counts and percentages [n (%)]. For univariate analyses, independent samples t-test was applied to normally distributed continuous variables; otherwise, the Wilcoxon rank-sum test was employed. The chi-square (χ²) test was used for categorical variables.

To identify and rank the importance of influencing factors, a random forest algorithm was implemented. Subsequently, the sliding windows sequential forward selection (SWSFS) method was utilized to determine the optimal number of variables corresponding to the minimum average out-of-bag (OOB) error rate. Variables with the highest importance scores and the lowest average OOB error rate were then included in a binary logistic regression model for further analysis. Statistical significance was set at a two-sided *P*-value < 0.05.

## 3 Results

### 3.1 Baseline characteristics

The median age of female participants was 32 years (interquartile range [IQR]: 29–37), with 37.26% classified as advanced maternal age (≥35 years). A majority of patients (60.67%) had a body mass index (BMI) within the normal range (18.5–24 kg/m²). The median duration of infertility was 3 years (IQR: 2–5). Detailed data is presented in **Table 1**.

**Table 1:** Basic information on the 7302 freeze-thawed embryo transfer (FET) cycles.

Variables		Non-clinical pregnancy (N=3937)	Clinical pregnancy (N=3365)	*Z*/*χ* ^2^	*P-value*	Total (N=7302)
Female age/years		34(30,39)	31(28,35)	-21.062	<0.001	32(29,37)
Female BMI/(kg/m^2^)	18.5~24	2372 (60.25%)	2058 (61.16%)	31.028	<0.001	4430(60.67%)
	<18.5	281 (7.14%)	313 (9.30%)			594(8.13%)
	24~28	1046 (26.57%)	743 (22.08%)			1789(24.50%)
	≥28	238 (6.05%)	251 (7.46%)			489(6.70%)
Male BMI/(kg/m^2^)	18.5~24	1694 (43.03%)	1506 (44.75%)	12.425	0.006	3200(43.82%)
	<18.5	95 (2.41%)	116 (3.45%)			211(2.99%)
	24~28	1590 (40.39%)	1256 (37.33%)			2846(38.98%)
	≥28	558 (14.17%)	487 (14.47%)			1045(14.31%)
Infertility years/years		3(2,6)	3(2,5)	-2.690	0.007	3(2,5)
Type of Infertility	Primary Infertility	1579 (40.11%)	1760 (52.30%)	108.747	<0.001	3339(45.73%)
	Secondary Infertility	2358 (59.89%)	1605 (47.70%)			3963(54.27%)

### 3.2 Clinical outcomes

A total of 7,302 frozen embryo transfer (FET) cycles were included in the analysis, resulting in 3,365 clinical pregnancies, corresponding to a clinical pregnancy rate (CPR) of 46.08%. When stratified by embryo stage, the CPR was 34.13% for cleavage-stage embryo transfers and 61.14% for blastocyst transfers. This difference was statistically significant (χ² = 528.973, *P* < 0.001). Additionally, 650 cycles resulted in miscarriage, yielding a miscarriage rate of 8.90%. Detailed data is presented in **Table 2**.

**Table 2:** 7302 frozen-thawed embryo transfer (FET) cycles transferred.

Variables		Non-clinical pregnancy (N=3937)	Clinical pregnancy (N=3365)	Total (N=7302)
Endometrial preparation protocols	Controlled ovarian hyperstimulation (COH)	63 (1.60%)	45 (1.34%)	108(1.48%)
	GnRHa* Down-regulation combined with HRT(GnRHa-HRT)	1438 (36.53%)	1322 (39.29%)	2760(37.80%)
	Hormone replacement therapy (HRT)	2300 (58.42%)	1888 (56.11%)	4188(57.35%)
	Natural cycle (NC)	136 (3.45%)	110 (3.27%)	246(3.37%)
AMH (ng/ml)		2.65(1.20,4.83)	4.03(2.34,6.86)	3.29(1.69,5.83)
Fertilization methods in fresh cycles	IVF*	3011 (76.48%)	2588 (76.91%)	5599(76.68%)
	ICSI*	815 (20.70%)	708 (21.04%)	1523(20.86%)
	IVF+RICSI	111 (2.82%)	69 (2.05%)	180(2.47%)
Pre-transplant endometrial thickness(mm)		8.00(7.00;9.00)	8.00(8.00,10.00)	8.00(7.00,9.00)
Type of embryo transfer	Cleavage embryo	2681 (68.10%)	1389 (41.28%)	4070(55.74%)
	Blastocyst	1256 (31.90%)	1976 (58.72%)	3232(44.26%)
High-quality cleavage embryo(pieces)	0	1918 (48.72%)	2156 (64.07%)	4074(55.79%)
	1	1159 (29.44%)	497 (14.77%)	1656(22.68%)
	2	856 (21.74%)	711 (21.13%)	1567(21.46%)
	3	4 (0.10%)	1 (0.03%)	5(0.07%)
Non-high quality cleavage embryo(pieces)	0	2558 (64.97%)	2811 (83.54%)	5369(73.53%)
	1	903 (22.94%)	382 (11.35%)	1285(17.60%)
	2	464 (11.79%)	165 (4.90%)	629(8.61%)
	3	12 (0.30%)	7 (0.21%)	19(0.26%)
High-quality blastocyst(pieces)	0	2845 (72.26%)	1506 (44.75%)	4351(59.59%)
	1	619 (15.72%)	796 (23.66%)	1415(19.38%)
	2	473 (12.01%)	1063 (31.59%)	1536(21.04%)
Non-high quality blastocyst(pieces)	0	3686 (93.62%)	3072 (91.29%)	6758(92.55%)
	1	206 (5.23%)	231 (6.86%)	437(5.98%)
	2	45 (1.14%)	62 (1.84%)	107(1.47%)
Endometrial thickness at date of transplantation(mm)		9.00 (8.00,10.00)	9.00 (8.00,11.00)	9.00(8.00,10.00)

*GnRHa, gonadotropin-releasing hormone agonist; IVF/ICSI, *in vitro* fertilization/intracytoplasmic sperm injection.

### 3.3 Ranking of influencing factors

Using the occurrence of clinical pregnancy as the dependent variable, a total of 15 potential predictors were evaluated for their relative importance via the random forest algorithm. These predictors included female age, female BMI, male BMI, endometrial preparation protocol, anti-Müllerian hormone (AMH) level, fertilization method in fresh cycles, type of infertility, duration of infertility, endometrial thickness prior to embryo transfer, type of transferred embryo, number of transferred high-quality cleavage-stage embryos, number of transferred low-quality cleavage-stage embryos, number of transferred high-quality blastocysts, number of transferred low-quality blastocysts, and endometrial thickness on the day of embryo transfer. Variables and naming conventions are shown in **Table 3**.

**Table 3:** Factors influencing and assigning values to clinical pregnancy outcomes.

Influencing factors	Assignment situation
Female age/years	——
Female BMI/(kg/m^2^)	1 = 18.5~24, 2=<18.5, 3 = 24~28, 4=≥28
Male BMI/(kg/m^2^)	1 = 18.5~24, 2=<18.5, 3 = 24~28, 4=≥28
Endometrial preparation protocol	1=COH, 2=GnRHa-HRT, 3=HRT, 4=NC
anti-Müllerian hormone (AMH)/(ng/ml)	——
Fertilization methods in fresh cycles	1=IVF, 2=ICSI, 3=IVF+RICSI
Type of Infertility	1= Primary Infertility, 2=Secondary Infertility
Infertility years/years	——
Pre-transplant endometrial thickness/mm	——
Type of embryo transfer	1=Cleavage embryo, 2= Blastocyst
Number of high-quality cleavage embryo/pieces	——
Number of non-high quality cleavage embryo/pieces	——
Number of high-quality blastocyst/pieces	——
Number of non-high quality blastocyst/pieces	——
Endometrial thickness at date of transplantation/mm	——
Clinical pregnancy	0=NO, 1=YES

**Figure 1:**
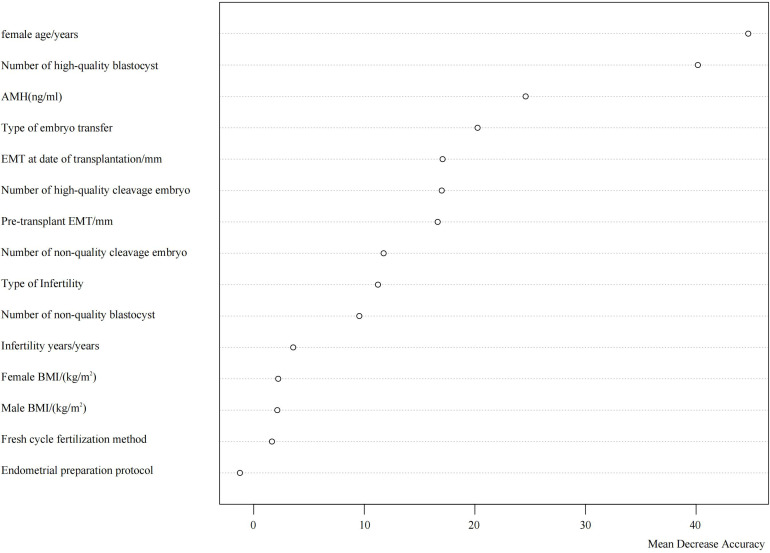
Ranking of importance of factors influencing clinical pregnancy outcome.

The analysis identified the top five most influential factors in descending order as follows: female age, number of high-quality blastocysts transferred, AMH level, type of transferred embryo, and endometrial thickness on the day of transfer. Their respective Mean Decrease Accuracy (MDA) values were 44.72, 40.16, 24.59, 20.24, and 17.08 (**Figure 1**).

### 3.4 SWSFS analysis

Based on the ranking of feature importance, a stepwise selection approach was employed to identify the most critical features. The window size was set to range from 1 to 15, with one feature added at each iteration. The average out-of-bag (OOB) error rate was used as the validation metric. At each iteration, the OOB error rate was calculated and recorded to evaluate the contribution of each additional feature to the model’s predictive performance. By progressively increasing the number of selected features, the optimal number of variables was determined as the point at which the model performance peaked.

**Figure 2:**
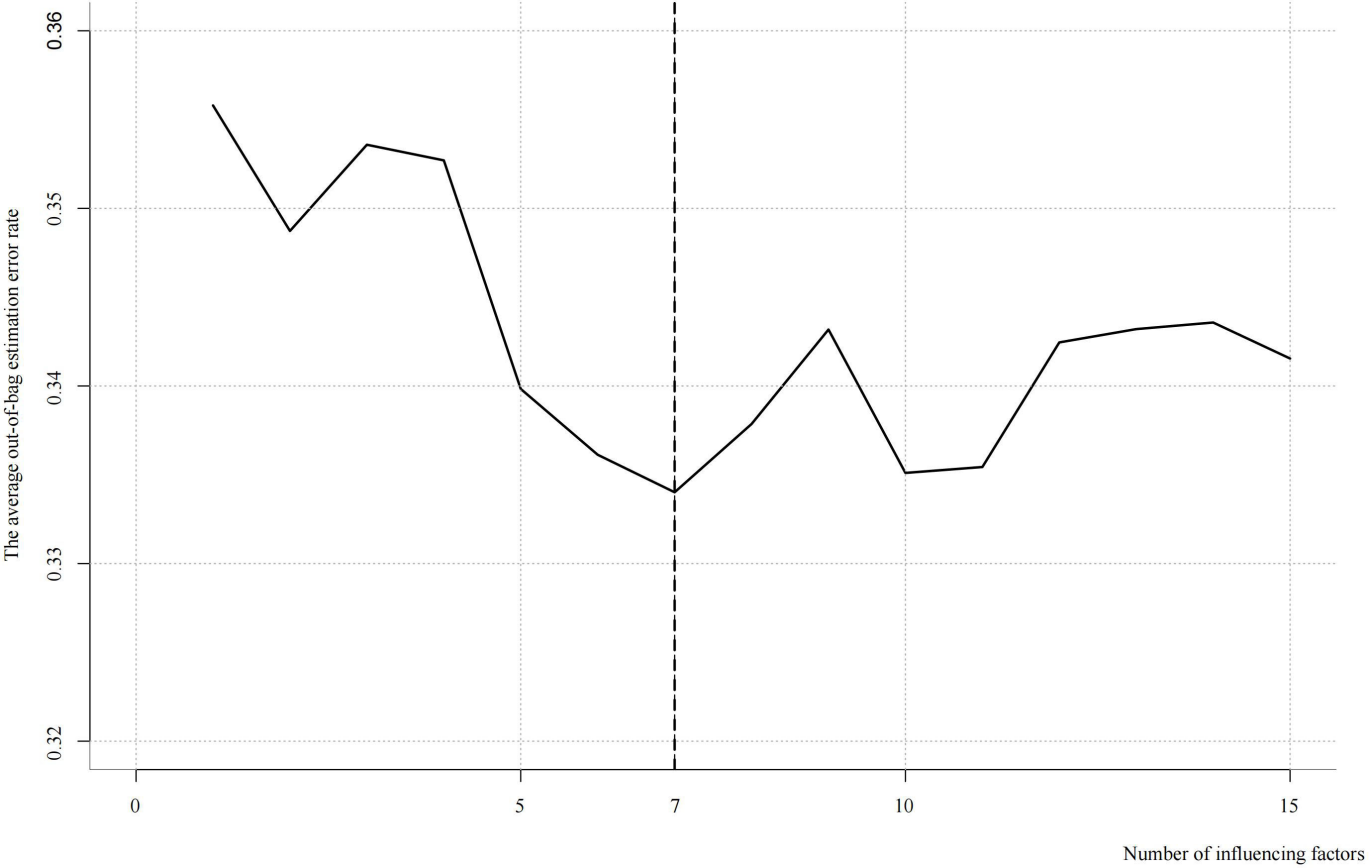
Results of SWSFS analysis.

The results indicated that the lowest average OOB error rate (0.33) was achieved when seven features were included. The top seven influential factors ranked by importance were: female age, number of high-quality blastocysts, anti-Müllerian hormone (AMH) level, type of transferred embryo, endometrial thickness on the day of transfer, number of high-quality cleavage-stage embryos, and endometrial thickness prior to transfer (**Figure 2**).

### 3.5 Regression analysis

**Figure 3:**
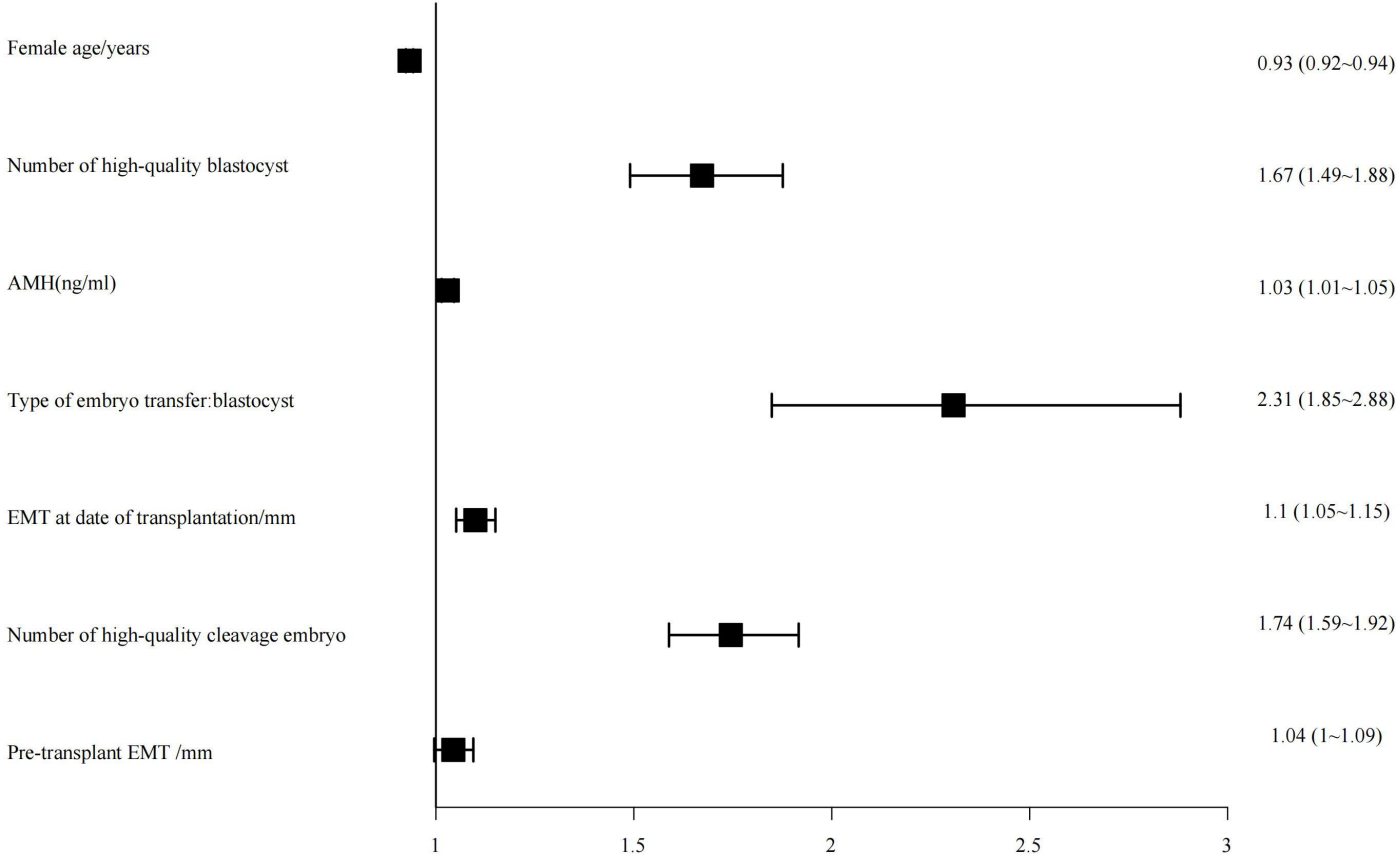
Forest plot of effects of factors influencing clinical pregnancy.

The top seven influential factors identified by the random forest algorithm were included as independent variables in a binary logistic regression model, with clinical pregnancy status as the dependent variable. The analysis revealed that maternal age, the number of high-quality blastocysts, anti-Müllerian hormone (AMH) levels, type of transferred embryo, endometrial thickness on the day of transfer, and the number of high-quality cleavage-stage embryos were all significantly associated with clinical pregnancy outcomes (**Figure 3**).

## 4 Discussion

In recent years, the rapid advancement of assisted reproductive technologies (ART) has been accompanied by continuous improvements in frozen embryo transfer (FET) techniques, leading to an increasing application of FET cycles in clinical practice (11). Despite the widespread adoption of FET owing to its advantages—such as enhancing cumulative pregnancy rates, minimizing embryo wastage, and reducing the incidence of moderate to severe ovarian hyperstimulation syndrome (OHSS)—the clinical outcomes of FET remain influenced by a complex interplay of multiple factors. Key determinants affecting clinical pregnancy rates (CPR) in FET cycles include maternal age, endometrial thickness, the number of embryos transferred, and the type of embryos used (8, 9, 12).

In the present study, the observed CPR was 46.08%, which is higher than that reported by Holschbach et al. (25.5%) (8) and Li Fan (46.0%) (13), yet lower than the 54.2% reported by Insogna et al. (14). Compared with the Chinese Society of Reproductive Medicine’s 2019 report indicating a CPR of 52.23% in FET cycles (15), our results reveal some discrepancy. Several factors may account for these differences. First, variations in patient selection criteria likely contributed; a subset of patients in our cohort were of advanced reproductive age with diminished ovarian reserve, resulting in fewer retrieved embryos and a lower proportion of high-quality embryos. Second, the quality of the frozen embryos varied—some patients underwent transfer of non-optimal embryos due to the absence of high-quality embryos, which could adversely affect pregnancy outcomes. Additionally, a minority of patients had a history of intrauterine adhesions requiring multiple hysteroscopic surgeries, with persistent thin endometrium prior to embryo transfer, which is known to compromise implantation potential. Since 2022, our center has adopted a predominantly single embryo transfer policy to reduce multiple pregnancy rates, which may also contribute to the observed CPR. Collectively, these factors could have impacted the clinical pregnancy outcomes observed in our cohort.

### 4.1 Age

Previous studies have consistently identified age as a critical determinant of successful assisted reproductive technology (ART) outcomes, including both fresh embryo transfer and frozen-thawed embryo transfer (FET) cycles (16, 17). In the present study, we observed a progressive decline in the likelihood of clinical pregnancy with advancing maternal age. Specifically, each additional year of age was associated with an approximate 7% decrease in the odds of achieving clinical pregnancy (OR: 0.93, 95% CI: 0.92–0.94). This finding aligns with the results of numerous prior investigations.

A systematic review and meta-analysis by Amerigo Vitagliano et al. (18) demonstrated a clear inverse relationship between increasing age and FET success rates. Similarly, research by Yi-Fei Sun et al. (19) identified maternal age as an independent predictor of clinical pregnancy outcomes in frozen embryo transfer cycles, with both implantation and clinical pregnancy rates significantly declining as age advances.

The detrimental impact of advanced maternal age on clinical pregnancy outcomes is likely multifactorial. Firstly, age-related ovarian decline results in a reduction of both the quantity and quality of oocytes, thereby decreasing the pool of viable high-quality embryos available for transfer (20). Secondly, endometrial receptivity diminishes with increasing age, which adversely affects embryo implantation rates (21). Moreover, advanced maternal age is frequently accompanied by comorbidities such as hypertension and diabetes, which may further compromise reproductive outcomes (22). Collectively, these factors contribute to the observed age-dependent reduction in clinical pregnancy success following FET.

### 4.2 Type and number of embryos transferred

In this study, the likelihood of achieving clinical pregnancy following blastocyst transfer was 2.31 times higher than that following cleavage-stage embryo transfer (95% CI: 1.85–2.88). Previous studies have established that blastocysts represent a more advanced stage of embryonic development with greater developmental potential, closely resembling the embryonic status during natural conception. Consequently, blastocysts exhibit enhanced implantation capacity and pregnancy potential. Furthermore, the extended culture period to the blastocyst stage allows for more intuitive and comprehensive assessment of embryo development, facilitating the selection of higher-quality embryos for transfer and thereby improving clinical pregnancy rates. Additionally, the timing of blastocyst transfer aligns more closely with the natural window of embryo implantation, which is physiologically advantageous for successful implantation (23).

Our findings also demonstrated that a greater number of high-quality blastocysts and cleavage-stage embryos transferred were positively associated with increased odds of clinical pregnancy (OR: 1.67, 95% CI: 1.49–1.88; OR: 1.74, 95% CI: 1.59–1.92, respectively), consistent with previous literature. Increasing the number of transferred embryos theoretically provides more opportunities for implantation, thus enhancing clinical pregnancy rates. However, expert consensus guidelines emphasize that while transferring three or more embryos may further increase clinical pregnancy rates (CPR), it substantially elevates the risk of multiple pregnancies—particularly higher-order multiples (three or more fetuses)—which are associated with significant maternal perinatal complications and adverse neonatal outcomes. Therefore, it is generally recommended that no more than two embryos be transferred per cycle to balance maximizing pregnancy success and minimizing the risks associated with multiple gestations (24, 25).

In the present cohort, patients undergoing transfer of three embryos were exceedingly rare, accounting for only 24 cases (0.33%) of frozen embryo transfer (FET) cycles. In clinical practice, individualized embryo transfer strategies should be adopted based on patient-specific factors and embryo quality to optimize clinical pregnancy outcomes while mitigating the risks of multiple pregnancies.

### 4.3 Anti-Müllerian hormone levels

Our findings indicate a positive correlation between anti-Müllerian hormone (AMH) levels and clinical pregnancy rate (CPR), consistent with previous studies by Xing Yu Sun (26), S. Ersahin (27), and others. AMH, a member of the transforming growth factor-beta (TGF-β) superfamily, is secreted by granulosa cells of small antral and pre-antral follicles within the ovarian cortex. It serves as a robust biomarker for assessing ovarian reserve and is recognized as a critical factor influencing clinical pregnancy outcomes following frozen embryo transfer (FET).

Several investigations, including those by Stylianos Vagios (28) and Xing Yu Sun (26), have identified AMH as an independent predictor of clinical pregnancy outcomes. However, contrasting evidence exists: some studies report that AMH has limited predictive value for clinical pregnancy following assisted reproduction (29, 30). These discrepancies highlight the need for further rigorous research into the prognostic utility of AMH in clinical pregnancy.

Several factors may contribute to the inconsistent findings regarding AMH’s predictive capacity. First, methodological variability in AMH measurement, including differences in assay platforms and reagents used across laboratories, can result in considerable inter-study variation (31). Second, heterogeneity in study populations, particularly across different age groups, may influence the association; for instance, AMH levels have shown a significant correlation with clinical pregnancy rates predominantly in women aged over 35 years, whereas this association appears weaker in younger cohorts (29). Third, study design and sample size limitations can affect the reliability of conclusions. Small sample sizes may undermine statistical power to detect meaningful relationships, and the nature of the study—retrospective versus prospective—also impacts data quality. Retrospective studies are susceptible to selection and information biases, whereas prospective designs typically afford a more accurate assessment of causal relationships.

In summary, while AMH remains a valuable biomarker of ovarian reserve and has potential utility in predicting FET outcomes, its definitive role as a predictor of clinical pregnancy requires further validation through large-scale, standardized, and prospective studies.

### 4.4 Endometrial thickness

Our findings demonstrate that endometrial thickness on the day of embryo transfer significantly influences clinical pregnancy rates (OR: 1.1, 95% CI: 1.05–1.15). The endometrium serves as the implantation site for the embryo, and an optimal endometrial thickness is a critical determinant for successful embryo implantation. Insufficient endometrial thickness can adversely affect the crosstalk between the embryo and the endometrium, thereby increasing the risk of implantation failure. As such, endometrial thickness on the transfer day is recognized as a vital indicator of uterine receptivity. A multicenter retrospective analysis reported that when endometrial thickness is below 6 mm, clinical pregnancy rates (CPR) decrease progressively with further thinning of the endometrium, whereas live birth rates tend to stabilize within the 7–10 mm range (32).

Previous studies highlight the complexity in defining a definitive cut-off value for endometrial thickness, which remains a contentious topic. Moreover, a thin endometrium has been implicated as a risk factor for adverse obstetric outcomes. Therefore, ongoing investigation into how varying degrees of endometrial thickness affect frozen-thawed embryo transfer (FET) outcomes is warranted, ideally through well-designed prospective studies (33, 34).

Endometrial thickness measured prior to embryo transfer also shows an association with clinical pregnancy outcomes, albeit with a smaller effect size (OR: 1.04, 95% CI: 1.00–1.09), suggesting a relatively modest impact compared to the thickness measured on the day of transfer. This indicates that while pre-transfer endometrial thickness exerts some influence on pregnancy outcomes, its role is comparatively limited. The pre-transfer endometrial thickness primarily reflects the adequacy of endometrial preparation. In cases where the endometrium is suboptimal before transfer, modifications to the preparation protocol—such as extending estrogen supplementation duration or adjusting hormone dosages—may be necessary to achieve an optimal endometrial thickness conducive to implantation (35).

### 4.5 Future perspectives

With the rapid advancement of the big data era and artificial intelligence (AI), hospitals are poised to harness large-scale data analytics and machine learning algorithms to conduct in-depth and precise analyses of extensive patient information, including age, ovarian function, and endometrial receptivity. By leveraging advanced image recognition technologies, embryo quality can be assessed with greater accuracy, while dynamic monitoring of parameters such as endometrial thickness and uterine blood flow can be implemented. The integration of AI-driven large models has the potential to transcend existing clinical paradigms, enabling the development of personalized and standardized protocols tailored to individual patients throughout the entire assisted reproductive process—from controlled ovarian stimulation, embryo culture, endometrial preparation, embryo transfer, to post-transfer monitoring. This approach aims to achieve precise control over embryo transfer procedures (36–39).

Future research should further elucidate the complex factors influencing frozen embryo transfer (FET) outcomes, including the immune microenvironment of the endometrium (40, 41) and hormonal profiles. Such insights will contribute to optimizing clinical strategies and improving reproductive success rates.

## 5 Study limitations

This study’s clinical data were exclusively obtained from the Department of Reproductive Genetics at the First Affiliated Hospital of Kunming Medical University, without inclusion of frozen-thawed embryo transfer (FET) cycles from other medical institutions, which may introduce admission bias. Secondly, the analysis was restricted to patients with complete clinical records who met the inclusion criteria, thereby limiting the generalizability of the findings to the hospital’s actual clinical pregnancy rates. Moreover, data on live birth outcomes remain incomplete at present, necessitating further longitudinal follow-up to enhance data comprehensiveness and enable more in-depth investigations. Additionally, this study represents a preliminary exploration of factors influencing clinical pregnancy outcomes following FET cycles. Future research should aim to conduct multicenter, large-sample studies as conditions permit, to promote the development of a multicenter FET database in the local setting. Efforts to develop more intelligent and precise predictive models are encouraged to achieve prospective and dynamic prediction of FET pregnancy outcomes, thereby providing robust support for safeguarding maternal and neonatal health and fulfilling reproductive goals.

## 6 Conclusions

In summary, female age, the number of high-quality blastocysts, anti-Müllerian hormone (AMH) levels, the transferred blastocyst(s), endometrial thickness on the day of embryo transfer, the number of high-quality cleavage-stage embryos, and endometrial thickness prior to transfer are all significant factors influencing clinical pregnancy outcomes in frozen-thawed embryo transfer (FET) cycles.

## Data Availability

The data analyzed in this study is subject to the following licenses/restrictions: Some information in the dataset may be restricted from use due to its sensitive nature. For specific usage of the dataset, please contact the corresponding author Limei He. Requests to access these datasets should be directed to Limei He, helimei9159@163.com.
